# Barely There But Existent: Angiotensin-Converting Enzyme Inhibitor–Induced Angioedema

**DOI:** 10.7759/cureus.94104

**Published:** 2025-10-08

**Authors:** Muhammad Asad Butt, Santosh Kumar, Syed Muaaz Sarwer, Daud Manna

**Affiliations:** 1 Anaesthesiology, Tipperary University Hospital, Clonmel, IRL; 2 Obstetric Anaesthesiology, Tipperary University Hospital, Clonmel, IRL

**Keywords:** ace inhibitors, airway swelling, difficult airway management, drug-induced angioedema, ecallantide, fresh frozen plasma for angioedema, history-taking, icatibant

## Abstract

We describe a 62-year-old female patient who presented to the Accident & Emergency (A&E) department with upper airway edema. Her past medical history included hypertension, obstructive sleep apnea, and ischemic heart disease. She had been previously hospitalized for a lower respiratory infection. On this occasion, she presented with upper airway swelling secondary to angiotensin-converting enzyme inhibitor (ACEI)-induced angioedema, requiring endotracheal intubation. Initial management in the A&E department, under differential consideration for anaphylaxis and Ludwig’s angina, included oxygen via a non-rebreather mask (NRM), intramuscular adrenaline, intravenous dexamethasone, nebulization with Pulmicort, and antibiotic coverage with ceftriaxone and metronidazole. Despite these measures, progressive airway edema necessitated intubation. The patient was subsequently transferred to a Level 4 hospital under the care of the maxillofacial team, managed conservatively, and maintained on ventilation until the edema resolved. Surgical intervention was deemed unnecessary. ACEI-induced angioedema is a rare but potentially life-threatening adverse effect of ACEIs, which are widely prescribed for hypertension, heart failure, and chronic kidney disease. A thorough medication history is essential for the timely recognition and management of this condition.

## Introduction

Hypertension and cardiac failure are managed with various medications, one of the most common being angiotensin-converting enzyme inhibitors (ACEIs). These drugs act by inhibiting the angiotensin-converting enzyme, thereby preventing the formation of angiotensin II. A known side effect of this class of medication is angioedema, a condition characterized by sudden swelling beneath the skin or mucous membranes due to a non-allergic reaction. This swelling may involve the face, lips, throat, or gastrointestinal tract [1]. Understanding the epidemiology and risk factors of ACEI-associated angioedema is essential for managing patients receiving these medications. ACEI-induced angioedema is associated with significant morbidity, including the need for endotracheal intubation, intensive care unit admission, and hospitalization, and fatal cases have been reported [2].

Here, we report the clinical presentation, anesthetic management, and outcome of a 62-year-old female patient who presented with perindopril-induced angioedema.

## Case presentation

This case report describes the management of a 62-year-old hypertensive woman who developed upper airway edema, later diagnosed as ACEI-induced angioedema. Her past medical history included hypertension, obstructive sleep apnea, and ischemic heart disease. She had also been hospitalized previously for a lower respiratory tract infection.

The patient presented with acute upper airway swelling that had worsened overnight. On history taking, there was no report of fever, jaw pain, trauma, dental infection, or insect bite. On examination, there was no rash or urticaria. She had difficulty swallowing, a swollen tongue, drooling, and progressive shortness of breath. Her respiratory rate was 25 breaths per minute, and oxygen saturation was 97% while on a non-rebreather mask (NRM). Chest auscultation revealed clear breath sounds, and she remained hemodynamically stable.

Initial management in the Accident & Emergency (A&E) department for suspected anaphylaxis and Ludwig’s angina included oxygen via a non-rebreather mask (NRM), intramuscular adrenaline 0.5 mg, intravenous dexamethasone 8 mg, nebulization with Pulmicort 0.5 mg, and antibiotic coverage with ceftriaxone 1 g and metronidazole 500 mg. Further review revealed that the patient had recently been prescribed bisoprolol, Trinomia (a combination of acetylsalicylic acid, atorvastatin, and perindopril, an ACEI), and clarithromycin (Klaricid) two to three days prior.

In the absence of urticaria, wheeze, hypotension, or bronchospasm, and given the patient’s ongoing ACEI therapy, ACEI-induced angioedema was suspected. There was no reduction in swelling or improvement in dysphagia and airway symptoms within 30 minutes of administering adrenaline, corticosteroids, and nebulization. Consequently, anaphylaxis-directed medications were discontinued, the ACEI was withheld, and management focused on vigilant airway monitoring.

The A&E team requested an anesthetic consultation for immediate airway management. On assessment, the patient had a Mallampati grade IV airway, a short neck, a BMI of 31 kg/m², and a known history of obstructive sleep apnea. Routine blood investigations were unremarkable (Table 1). The anesthetic consultant formulated an anticipated difficult airway plan, which included calling for assistance, preparing resuscitation drugs, and ensuring a difficult airway trolley was readily available, with a plan for awake fibreoptic intubation (AFOI). The patient was transferred to the operating room, where inhalational induction with sevoflurane was initiated while maintaining spontaneous respiration and administering incremental doses of midazolam. Intubation was attempted using a size 3 McGrath video laryngoscope blade. The first attempt was unsuccessful due to marked glottic edema. On the second attempt, tracheal intubation was successfully achieved using a 6.0 mm cuffed oral endotracheal tube (COETT) railroaded over a bougie and secured at 26 cm. The significant tongue swelling necessitated securing the COETT at this position.

**Table 1:** Laboratory findings MCV: mean corpuscular volume, BUN: blood urea nitrogen, CRP: C-reactive protein.

Laboratory values	Patient values	Reference values
White blood cell count	9	3.5-10.5 K/µL
Hemoglobin	14.5	12-16 g/dL
Platelet	190	150-450 K/µL
MCV	90.4	80-100 µm^3^
Neutrophils	6.43	1.5-7.5 K/µL
Monocytes	0.5	0-0.8 × 10^3^/µL
Eosinophils	0.08	0-0.5 × 10^3^/ µL
Creatinine	1	0.6-1.10 mg/dL
BUN	6	6-20 mg/dL
Potassium	4.3	3.5-5.1 mmol/L
CRP	74	<1 mg/dL

**Figure 1:**
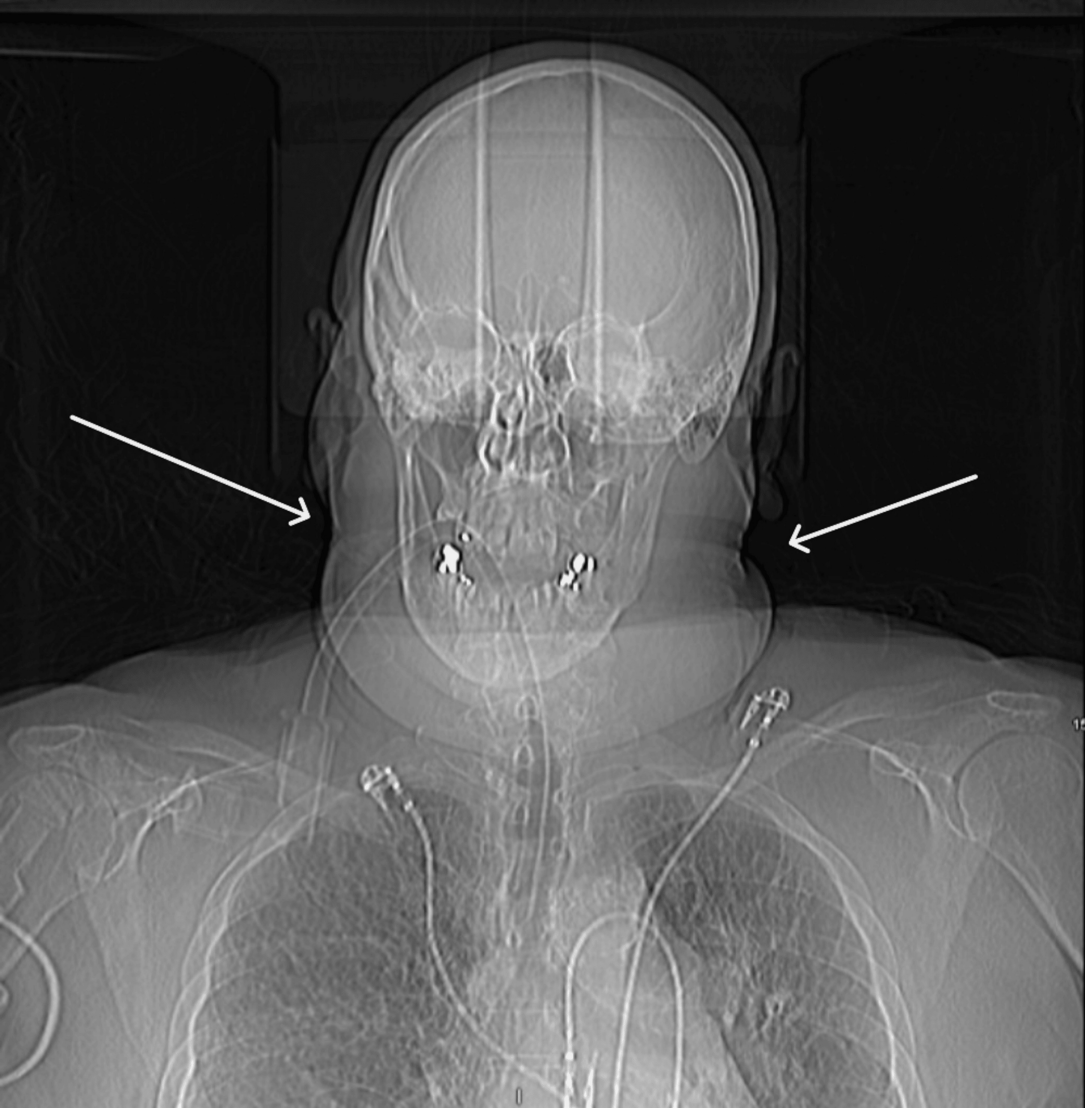
Computed tomography scan image showing significant neck swelling (arrows)

**Figure 2:**
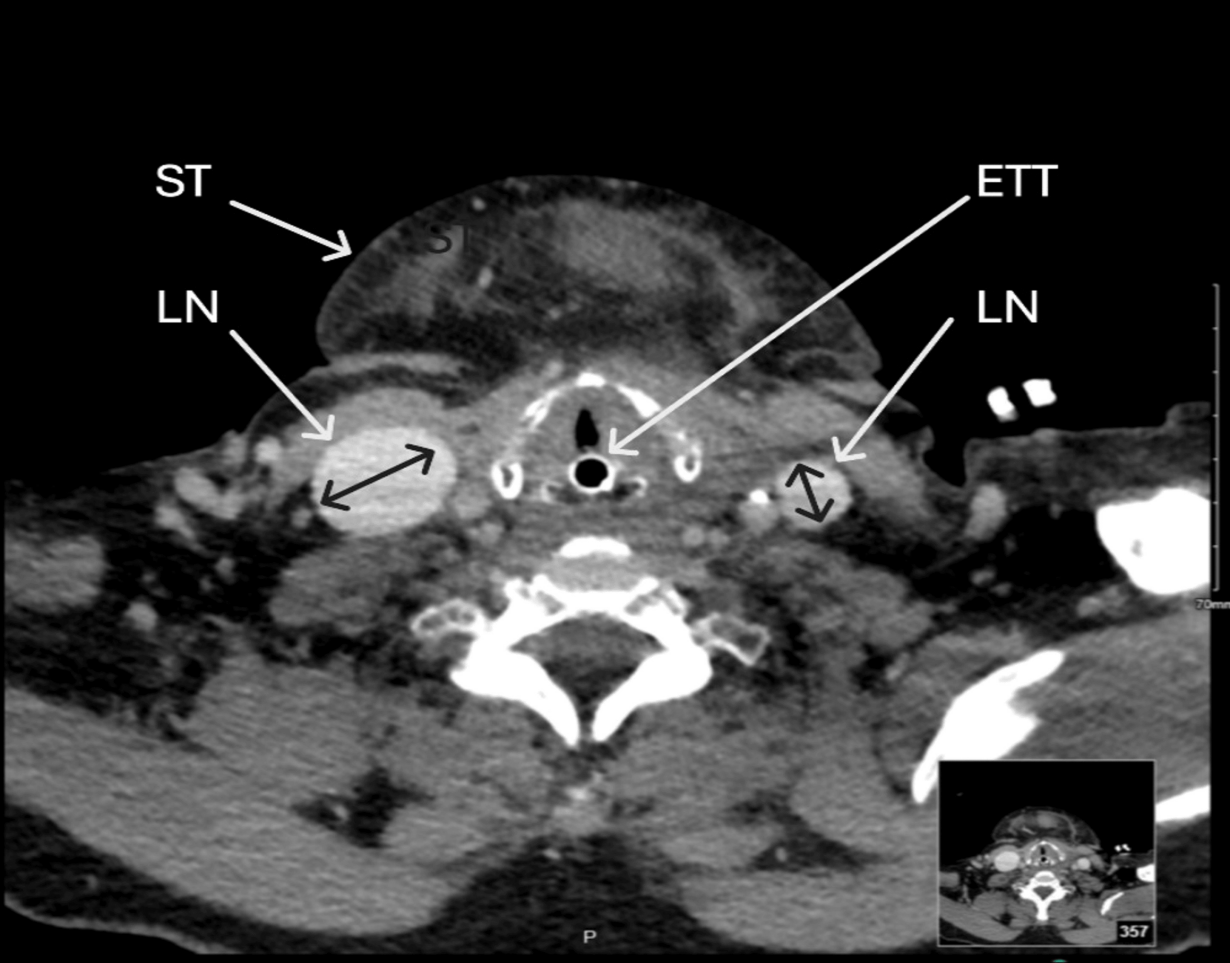
Computed tomography of the head and neck (axial view) Endotracheal tube (ETT) in situ; dental implant reflection noted. There is significant bilateral submandibular lymphadenopathy (LN) with extensive submental lymphadenopathy and soft tissue (ST) edema. An associated abscess cannot be excluded. Edema is also seen at the base of the tongue. Specialist review is required.

A computed tomography (CT) scan (Figure 1 and Figure 2) performed after securing the airway revealed significant lymphadenopathy and raised the possibility of a submandibular abscess. The case was discussed with the maxillofacial surgery department at a Level 4 hospital, which accepted the patient for transfer. No surgical intervention was required, as further evaluation ruled out an infectious etiology or abscess. The patient remained intubated for 72 hours and was managed according to intensive care unit protocols. After 72 hours, the swelling had markedly subsided, allowing a successful weaning trial and extubation. Perindopril, identified as the offending drug, was discontinued following the pharmacist’s recommendation. The patient remained hospitalized for two weeks post-extubation for management of comorbidities, after which she was transferred back to the referring hospital and subsequently discharged home with a good outcome.

## Discussion

ACEI-induced angioedema is a rare but serious adverse effect of medications commonly prescribed for hypertension, heart failure, and chronic kidney disease. It presents as sudden soft tissue swelling, most often involving the head and neck region. Unlike other forms of angioedema, it typically occurs without itching or urticaria, which can delay recognition and diagnosis. In some cases, gastrointestinal involvement may occur, leading to abdominal pain, vomiting, and diarrhea.

The reported incidence ranges from 0.1% to 0.7%, with certain populations at higher risk [3]. Individuals of African descent are particularly susceptible, with incidence rates up to five times greater than in other populations [4]. The most significant risk factors identified are Black race and ACEI use [4]. Genetic predisposition may also contribute to the development of this condition.

The underlying mechanism involves the accumulation of bradykinin, a peptide that promotes vasodilation and increases vascular permeability, resulting in localized swelling. Normally, the angiotensin-converting enzyme degrades bradykinin; however, ACEIs inhibit this process, leading to elevated bradykinin levels and subsequent angioedema [5]. ACEI-related angioedema differs from typical allergic reactions, as it is not histamine-mediated and does not involve an IgE-dependent immune response [6].

Diagnosis is primarily clinical, as no specific laboratory test confirms ACEI-induced angioedema. Nonetheless, investigations may aid in excluding alternative causes of the patient’s presentation [7].

Management of ACEI-induced angioedema begins with the immediate discontinuation of the offending drug and the provision of general supportive care. Symptoms typically resolve spontaneously within two to three days after stopping the medication [8]. Since this condition is not histamine-mediated, standard treatments for allergic reactions, such as corticosteroids, antihistamines, and epinephrine, are usually ineffective [9]. Some case reports suggest that therapies including fresh frozen plasma, C1-esterase inhibitor concentrate, ecallantide, and icatibant may shorten symptom duration; however, these observations have yet to be validated by clinical trials [10,11].

For patients with a history of ACEI-induced angioedema, alternative antihypertensive agents such as angiotensin II receptor blockers (ARBs), calcium channel blockers, and other classes are preferred, as they do not affect bradykinin metabolism and carry a lower risk of recurrence [5]. Although ARBs are generally safer, careful monitoring remains essential [12].

Angioedema can progress rapidly, making airway assessment the top priority. If the airway is compromised, prompt intervention following local anaphylaxis or airway management protocols is essential, which may include endotracheal intubation or, if necessary, a surgical airway [13]. Visualization using nasopharyngeal laryngoscopy is recommended for patients presenting with stridor or hoarseness to assess the extent of laryngeal involvement. Intubation is more often required when the swelling involves the pharynx or larynx rather than being confined to the lips or face. A useful clinical rule is that swelling anterior to the teeth typically responds to medical therapy, while swelling posterior to the teeth often necessitates airway intervention. The likelihood of requiring intubation also increases with advancing age.

When intubation is required, nasopharyngeal or endotracheal routes are preferred. Bradykinin-mediated angioedema commonly affects the lips and tongue, often obstructing the oral route, while the nasal passage is typically spared, rendering supraglottic devices unsuitable. Awake nasopharyngeal intubation is considered the safest approach; however, the edematous tissues are highly susceptible to trauma, and airway manipulation may exacerbate swelling. Therefore, airway management should be undertaken only by clinicians experienced in nasopharyngeal intubation and prepared for an emergency surgical airway if necessary. Intensive care unit monitoring is recommended for patients with lingual edema and is mandatory for those with laryngeal involvement.

## Conclusions

Angioedema caused by ACEIs is an uncommon but potentially life-threatening adverse effect. Because of the widespread use of these medications, clinicians must maintain a high index of suspicion. A thorough review of the patient’s medication history is crucial, as the diagnosis is largely clinical and relies on excluding other causes. This case highlights the need for caution when prescribing perindopril and other ACEIs. Although ARBs are generally considered safer alternatives, they can also trigger angioedema, particularly in patients with a prior episode related to ACEIs. Patients should be informed of this rare but serious side effect, and close monitoring is essential when symptoms appear, as airway compromise and respiratory failure may develop rapidly.
